# Steroids as Central Regulators of Organismal Development and Lifespan

**DOI:** 10.1371/journal.pbio.1001307

**Published:** 2012-04-10

**Authors:** Siu Sylvia Lee, Frank C. Schroeder

**Affiliations:** 1 Department of Molecular Biology and Genetics, Cornell University, Ithaca, New York, United States of America; 2 Boyce Thompson Institute and Department of Chemistry and Chemical Biology, Cornell University, Ithaca, New York, United States of America

## Abstract

Larvae of the nematode *Caenorhabditis elegans* must choose between reproductive development and dauer diapause. This decision is based on sensing of environmental inputs and dauer pheromone, a small molecule signal that serves to monitor population density. These signals are integrated via conserved neuroendocrine pathways that converge on steroidal ligands of the nuclear receptor DAF-12, a homolog of the mammalian vitamin D receptor and liver X receptor. DAF-12 acts as the main switch between gene expression programs that drive either reproductive development or dauer entry. Extensive studies in the past two decades demonstrated that biosynthesis of two bile acid-like DAF-12 ligands, named dafachronic acids (DA), controls developmental fate. In this issue of *PLoS Biology*, Wollam et al. showed that a conserved steroid-modifying enzyme, DHS-16, introduces a key feature in the structures of the DAF-12 ligands, closing a major gap in the DA biosynthesis pathway. The emerging picture of DA biosynthesis in *C. elegans* enables us to address a key question in the field: how are complex environmental signals integrated to enforce binary, organism-wide decisions on developmental fate? Schaedel et al. demonstrated that pheromone and DA serve as competing signals, and that a positive feedback loop based on regulation of DA biosynthesis ensures organism-wide commitment to reproductive development. Considering that many components of DA signaling are highly conserved, ongoing studies in *C. elegans* may reveal new aspects of bile acid function and lifespan regulation in mammals.

*C. elegans* normally goes through a simple life cycle: from egg, through four larval stages, to reproductive adult. However, under adverse environmental conditions, these worms enter an alternate third larval stage termed dauer. Compared to normal third stage larvae, dauer larvae have dramatically different metabolism and physiology, and distinct morphology and behavior [1], which confer greatly increased stress resistance and facilitate dispersal. When environmental conditions improve, *C. elegans* exit dauer and resume reproductive development. The dauer stage is generally considered as “non-aging,” as dauers can persist for months before recovering to develop into a reproductive adult that lives the normal lifespan of a few weeks. Not surprisingly, recent findings suggest that re-activation of some of the molecular signature of dauer later in life contributes to prolonged longevity in *C. elegans* [2].

## The Dauer Pheromone

The major environmental cues that regulate dauer formation include food, temperature, and a small molecule signal constitutively produced by the worms: the dauer pheromone [3],[4]. Elevated pheromone levels caused, for example, by over-crowding are a strong cue for dauer entry. Extensive studies in the past few decades have revealed a complex network of signaling pathways that regulate the decision on reproductive maturation versus dauer diapause. Early mutagenesis screens identified a large number of mutants that enter or exit dauer inappropriately, commonly referred to as “*daf*” (dauer formation) mutants, many of which turned out to represent components of highly conserved signaling pathways. Environmental cues are perceived by a number of sensory neurons [5],[6], which are coupled to two neuroendocrine signaling pathways: the *daf-2* insulin/insulin-like growth factor signaling (IIS) pathway and the *daf-7* TGF-β signaling pathway [7]–[9].

## Activation of Nuclear Hormone Receptor DAF-12 Serves as a Key Developmental Switch

Genetic evidence revealed *daf-12*, a nuclear hormone receptor with homology to mammalian vitamin D receptor and LXR, as the main switch on which several upstream dauer signaling pathways converge. *daf-12* acts as a ligand-dependent transcription factor that locks in the final decision on reproductive versus dauer development [5],[8],[10]. In addition, the P450 *daf-9* was found to act upstream of *daf-12* antagonizing dauer entry [11],[12]. Based on a large body of evidence it was concluded (1) that DAF-12 activity is controlled by a steroidal ligand whose biosynthesis relies in part on *daf-9* and (2) that these steroidal DAF-12 ligands drive reproductive development, whereas unliganded DAF-12 promotes entry into the dauer stage [12],[13]. Earlier studies revealed DAF-9 expression in the pair of neuron-like XXX cells, suggesting XXX represent foci in which upstream DAF-2 and DAF-7 signaling integrate with the biosynthesis of DAF-12 ligand(s) [12]–[14].

**Figure 1:**
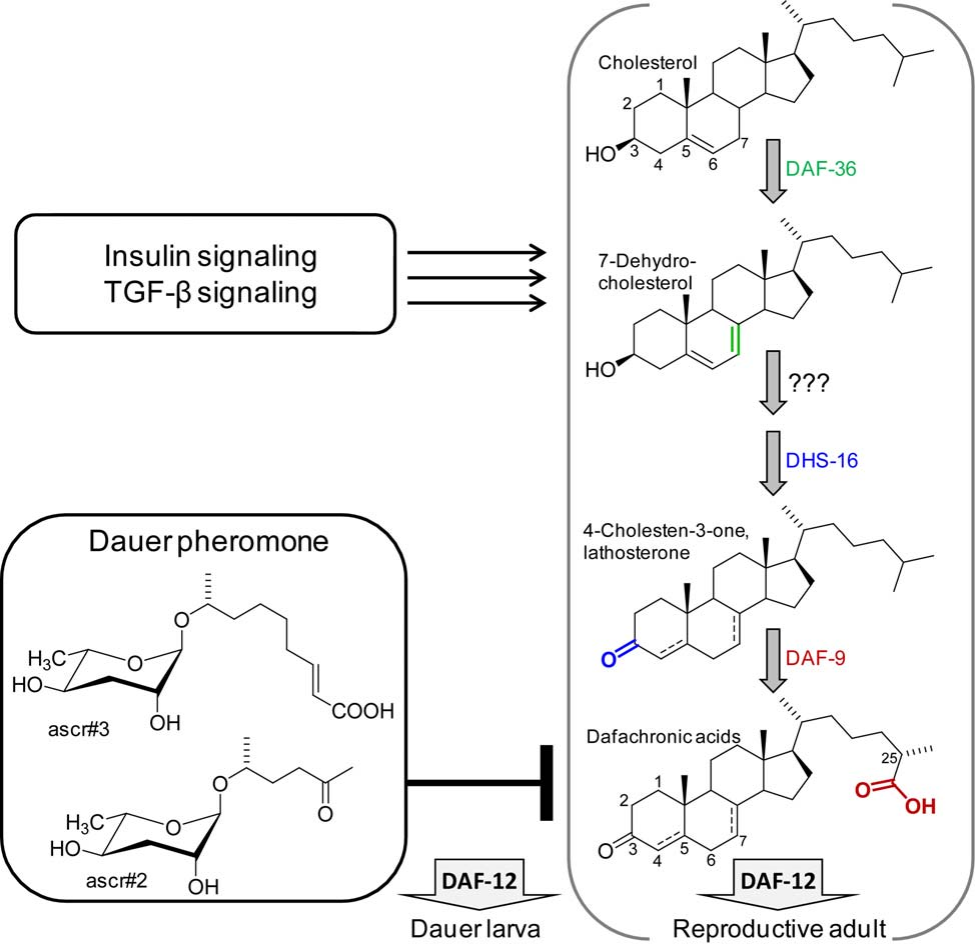
Dafachronic acid biosynthesis controls the decision between dauer and reproductive development. Insulin signaling and TGF-β signaling promote DA biosynthesis, driving development to reproductive adults, whereas dauer pheromone (ascarosides) abolish DA biosynthesis, resulting in unliganded DAF-12 and dauer formation.

Until recently surprisingly little was known about the role of small molecules in these pathways. Not until 2005, more than 20 years after its initial discovery by Golden and Riddle, was the first component of the dauer pheromone identified. In the past few years, it has become apparent that the “dauer pheromone” in fact constitutes a multi-component mixture consisting of at least 10 different, but chemically and biosynthetically related compounds. All dauer pheromone components consist of the dideoxysugar ascarylose and include a variable fatty-acid derived side chain and occasionally carry other substituents (for example, ascr#2 and ascr#3 in Figure 1) [15]–[19]. In part, identification of the dauer pheromone may have been delayed because of its complex composition, which makes activity-guided isolation of dauer-inducing compounds from the highly complex *C. elegans* metabolite extracts more difficult. Subsequent studies have shown that many of the dauer pheromone components also affect *C. elegans* behavior, often at concentrations far below those that induce dauer [18],[19].

Consistent with earlier studies highlighting sensory neurons responsible for detecting environmental cues, recent progress has identified specific sensory neurons and G-protein-coupled receptors involved in ascaroside perception [20],[21]. However, the mechanisms by which ascarosides effect the pervasive organismal changes that are associated with abandoning reproductive development and entering the dauer diapause have only partly been understood.

## Dafachronic Acids Are Ligands of DAF-12

As outlined above, DAF-12 acts as a central switch governing the decision between reproductive development and dauer. As in the case of the dauer pheromone, the identity of the small molecule ligands of DAF-12 only became apparent long after the main genetic components of the dauer signaling network had been characterized. In 2006, Motola et al. [22] described two bile acid-like compounds, named dafachronic acids (DA), as the endogenous ligands of DAF-12. The two dafachronic acids, bearing a double bond either in position 4 or 7 of the steroid skeleton (Δ^4^- and Δ^7^-dafachronic acids, Figure 1) were shown to activate DAF-12 *in vitro* and, as expected, were found to promote reproductive development [22]. Subsequent studies showed that DAs also regulate adult lifespan in *C. elegans*, and that DA signaling is widely conserved among nematodes, including parasitic nematodes whose infective larval stages correspond to *C. elegans* dauer larvae [23],[24].

*C. elegans* developmental fate and lifespan thus ultimately depend on the DA biosynthetic pathway. Therefore, elucidation of this pathway and its regulation is of central importance for understanding the mechanisms by which pheromone and other environmental signals direct *C. elegans* life history. It is known that cholesterol is required for nematode viability chiefly because it serves as starting material for DA biosynthesis, but only two steps of the DA biosynthetic pathway have been elucidated: in the first step of DA biosynthesis the Rieske-like oxidase DAF-36 converts cholesterol into 7-dehydrocholesterol [25], whereas in the last step of DA biosynthesis P450 DAF-9 add the carboxylic acid moiety to the side-chain terminus [22] (Figure 1). In particular, the origin of the characteristic 3-keto group, which is essential for DA activity, has remained unclear. Previous work suggested that HSD-1, a putative 3-β-hydroxysteroid dehydrogenase (3β-HSD) homolog, may introduce the 3-keto group, though the precise role of this enzyme in DA biosynthesis was not determined [26],[27].

## Elucidation of Dafachronic Acid Biosynthesis

In this issue of *PLoS Biology*, Wollam et al. performed a genome-wide RNAi screen looking for enhancers of a weak dauer phenotype of *daf-36* mutant worms. These mutant worms have reduced DA levels just about sufficient for normal reproductive development, and therefore RNAi knockdown of any additional components of the DA biosynthesis pathway should lead to dauer phenotypes. This screen revealed the short-chain dehydrogenase/reductase (SDR) *dhs-16* as an enhancer of the *daf-36* dauer phenotype. Genetic epistasis experiments showed that *dhs-16* acts downstream of insulin/IGF-1 and TGF-β signaling pathways, but upstream of DAF-12, indicating that DHS-16 participated in DA biosynthesis. To determine the exact role of DHS-16 in DA biosynthesis, Wollam et al. conducted a series of sterol feeding experiments, which showed that 3-keto steroids rescue the *dhs-16* phenotype, whereas the corresponding 3-hydroxy steroids were without effect. These results suggested that DHS-16 has 3β-dehydrogenase activity, converting lathosterol, and perhaps other 3-hydroxy sterols, into the corresponding 3-keto derivatives (Figure 1).

The identification of DHS-16 as a 3β-HSD introducing the characteristic 3-keto functionality closes a major gap in the DA biosynthesis pathway. Although DHS-16 is not orthologous to known mammalian 3β-HSDs, which appear to rely on a different chemical mechanism for introducing the 3-keto functionality, there are several mammalian short-chain dehydrogenases, for example the liver-expressed SDR-O/SDR9C7, that are close homologs. The finding that DHS-16 has 3β-HSD activity and participates in the biosynthesis of bile acid-like molecules that control *C. elegans* development and lifespan may suggest that its mammalian homologs serve analogous roles in bile acid metabolism. If confirmed, such close homology may suggest the intriguing possibility that bile acid-like steroids regulate mammalian lifespan, which to date has not been comprehensively explored.

## Detecting Dauer-Inducing Environmental Signals

As a clearer picture of DA biosynthesis emerges, tools become available to address a most fascinating aspect of life history decisions: how are organism-wide developmental changes coordinated? The decision between entering the dauer diapause and reproductive development must rely on robust temporal and spatial coordination mechanisms to avoid partial phenotypes. To uncover these mechanisms, Schaedel et al. first investigated the time points at which developing *C. elegans* larvae integrate environmental signals before committing to dauer or reproductive development. Using dauer pheromone and DA as opposing stimuli, Schaedel et al. established that the decision can be affected by DA and pheromone only during precisely defined time windows prior to ultimate commitment to life cycle fate. Furthermore, it became apparent that dauer pheromone and DA are directly competing stimuli: higher dauer pheromone concentrations require higher DA levels to prevent commitment to dauer entry. Unexpectedly, *daf-9* null mutants, which are incapable of producing DA, respond differently than wild type worms to conditions that are near the dauer-inducing threshold: whereas cohorts of wild type larvae exposed to moderate dauer pheromone concentrations develop into a mixture of dauer larvae and fully developed adults, *daf-9* mutants exposed to low concentrations of added DA develop into animals displaying a range of intermediate phenotypes, including worms that bypass the dauer stage yet exhibit an abnormal phenotype. That these intermediate phenotypes occur almost exclusively in *daf-9* worms suggests that organism-wide commitment to either dauer or reproductive development involves direct regulation of *daf-9*.

**Figure 2:**
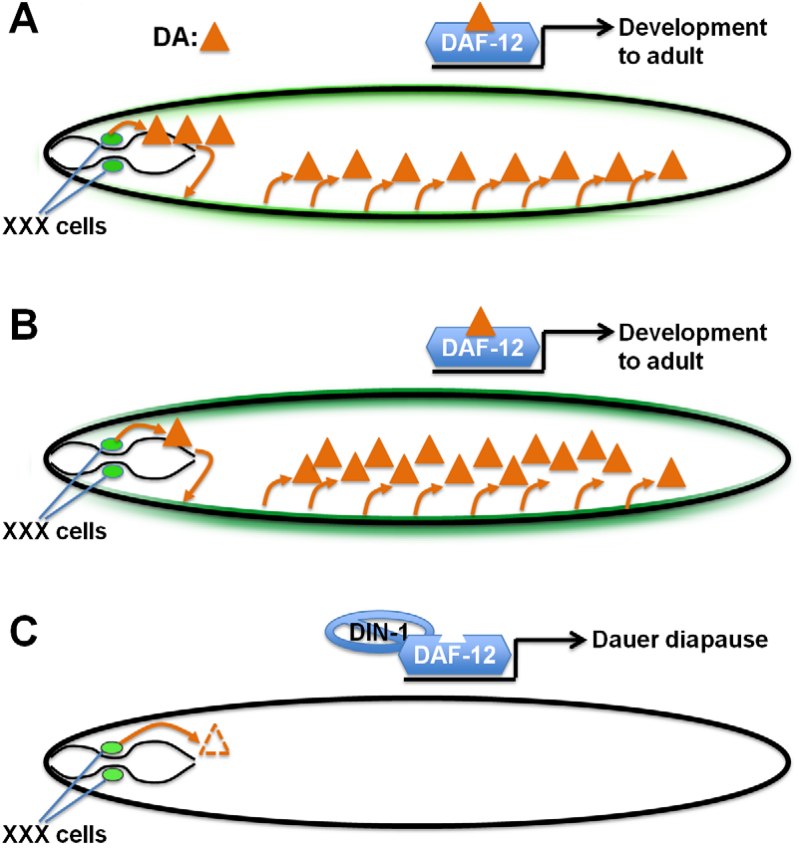
Dafachronic acid feedback directs organism-wide commitment to specific developmental fates. (A) Under favorable conditions, XXX-produced DA is amplified via *daf-9* expression in hypodermal cells (green). (B) Under marginal conditions, increased hypodermal *daf-9* expression maintains sufficient DA levels to prevent intermediate phenotypes. (C) Under unfavorable conditions, low dafachronic acid production in the XXX cells is insufficient to turn on hypodermal DA production. The unliganded DAF-12 then recruits the corepressor DIN-1, promoting dauer development.

Using transgenic *daf-9* null worms expressing GFP under the control of the *daf-9* promotor, Schaedel et al. showed that in the absence of DA, *daf-9* transcription is turned on only in the XXX cells, whereas intermediate DA concentrations resulted in a dramatic increase in its expression in hypodermal cells during the time window the larva must decide between dauer and reproductive development (Figure 2). Somewhat counter-intuitively, even higher concentration of externally added DA resulted in *decreased* hypodermal *daf-9* transcription. The finding that *daf-9* is expressed constitutively in the XXX cells whereas hypodermal *daf-9* expression is regulated by DA suggested that DA produced by XXX may promote *daf-9* expression in hypodermal cells. To test this hypothesis, Schaedel et al. laser-ablated the XXX cells in larvae expressing a DAF-9 protein tagged with GFP. They observed that almost all XXX-ablated worms enter the dauer stage, instead of developing into normal adults, and lacked DAF-9 expression in hypodermal cells. Dauer formation in these worms could be fully rescued by addition of DA, without forming any of the intermediate phenotypes seen for the *daf-9* null mutants with DA. Taken together, these results suggest that the XXX cells act as a source of DA that triggers strong additional DA biosynthesis in the hypodermis, locking in organism-wide commitment to reproductive adult development. In response to favorable conditions this feedback loop maintains DA levels high enough to continue reproductive development, whereas unfavorable conditions appear to trigger reduced DA production in the XXX cells, resulting in complete shutdown of DA biosynthesis to ensure organism-wide initiation of the transition into dauer.

## Longer Term Considerations and Longevity

The study of DA biosynthesis and its regulation thus revealed an elegant self-regulatory loop enforcing a binary decision for dauer or reproductive development. However, several aspects of this regulatory network remain to be uncovered. Mechanistically it is unclear how under favorable conditions, when DA levels are high, hypodermal *daf-9* is kept at an intermediate level, whereas under marginal conditions, when DA levels are intermediate, hypodermal *daf-9* becomes highly upregulated (Figure 2). To address this question, it will be necessary to measure actual DA levels in worms at different time points under different conditions, as DAF-9 is just one of several enzymes in the DA biosynthetic pathway. Additionally, the possibility that different DAs have different functions must be explored.

Schaedel et al. further show that pheromone and DA act as competing signals. Presumably, higher levels of pheromone modulate upstream DAF-2 and DAF-7 signaling that eventually affects expression of DA biosynthesis genes. Similarly, the molecular mechanisms of how food-derived signals and temperature sensing contribute to the dauer decision remains to be clarified. It is likely that these factors represent environmental inputs that are sensed by upstream signaling, but it is also possible that the food signal represents a metabolic input that influences dauer decision by upregulating DAF-2/insulin signaling.

Lastly, the DA biosynthetic pathway is still far from complete. Wollam et al. showed that HSD-1 is not involved in Δ^7^-DA biosynthesis as previously hypothesized. Nonetheless, the *hsd-1* dauer phenotype [26],[27] strongly suggests that it contributes to DA biosynthesis in some manner. By extension, the *C. elegans* genome contains many additional HSD, DHS, and CYP450, some of which likely participate in DA biosynthesis. If additional components of DA exist, then possible tissue specificity of their biosynthesis and action will be important questions for future investigation.

We should note that the DA biosynthetic pathway has a key role in determining longevity in *C. elegans*, particularly in response to germline signals. Furthermore, the pheromone responsive pathways DAF-2 and DAF-7 are both key mediators of longevity [28],[29]. Therefore, it appears that pheromone and DA not only regulate a fate choice during development but continue to modulate physiology through adulthood and have a major impact on longevity. In this regard, IIS and TGFβ in mammals are strongly implicated to be important for aging and age-dependent pathologies. Considering the highly conserved nature of both the biosynthetic genes and the chemical nature of DAs as bile acids, ongoing studies in *C. elegans* may reveal new aspects of bile acid function in mammals. Whether bile acids will turn out to be important for mammalian aging and aging pathologies remains an interesting area for future investigation.
